# A Data-Driven Hybrid Three-Stage Framework for Hospital Bed Allocation: A Case Study in a Large Tertiary Hospital in China

**DOI:** 10.1155/2019/7370231

**Published:** 2019-05-02

**Authors:** Li Luo, Jialing Li, Xueru Xu, Wenwu Shen, Lin Xiao

**Affiliations:** ^1^Business School of Sichuan University, No. 24 South Section 1, Yihuan Road, Chengdu, China; ^2^West China Hospital of Sichuan University, No. 17 People's South Road, Chengdu, China

## Abstract

Beds are key, scarce medical resources in hospitals. The bed occupancy rate (BOR) amongst different departments within large tertiary hospitals is very imbalanced, a situation which has led to problems between the supply of and the demand for bed resources. This study aims to balance the utilization of existing beds in a large tertiary hospital in China. We developed a data-driven hybrid three-stage framework incorporating data analysis, simulation, and mixed integer programming to minimize the gaps in BOR among different departments. The first stage is to calculate the length of stay (LOS) and BOR of each department and identify the departments that need to be allocated beds. In the second stage, we used a fitted arrival distribution and median LOS as the input to a generic simulation model. In the third stage, we built a mixed integer programming model using the results obtained in the first two stages to generate the optimal bed allocation strategy for different departments. The value of the objective function, *Z*, represents the severity of the imbalance in BOR. Our case study demonstrated the effectiveness of the proposed data-driven hybrid three-stage framework. The results show that *Z* decreases from 0.7344 to 0.0409 after re-allocation, which means that the internal imbalance has eased. Our framework provides hospital bed policy makers with a feasible solution for bed allocation.

## 1. Introduction

The inherent difference between limited resources for healthcare and steadily increasing demands occurs all over the world and is particularly serious in developing countries. According to a research report from the World Health Organization (WHO) and World Bank Groups, at least 400 million people worldwide cannot receive one or more basic health services [1].

This differential is particularly apparent with respect to bed resources. Although hospital bed numbers have increased greatly in recent years, this increase cannot cope with the growth rate of admission demand in China. According to the report “Statistical Communique on the Development of China's Health and Family Planning Program 2016” [2], the number of hospitalizations across the nation's medical and health institutions was 227.28 million and the annual hospitalization rate was 16.5%. There were 7.410 million beds in medical institutions across the country. Amongst all of the medical institutions, China's large tertiary hospitals, classed as Class III according to the classification standards (Appendix), are facing the most serious imbalance between admissions and bed resources (Table 1). The number of hospital beds and individuals hospitalized in Class III hospitals increased to 2,213,718 and 76,860,000, respectively. Bed occupancy rates (BORs) reached 98.8% in 2016.

We found that the imbalance between supply and demand in large tertiary (Class III) hospitals is greater than that in the other two classes of hospitals. Hospital managers urgently need to find solutions to alleviate bed shortages. Hospital administrators typically address this issue in two ways: by improving utilization of existing beds or by expanding capacity. The first involves the complex task of strategically allocating the proper amount of beds for each set of care types. A hospital that is unable to find an optimal allocation may acquire additional beds. However, an expansion, which is desirable for one hospital, may not be advantageous from the perspective of the public planner [3] and may even have some negative consequences, such as doctor and nurse work overloads, decreases in medical care quality, and aggravation of medical conflicts [4]. Therefore, from the perspective of the sustainability of medical resources, the best option is to improve the utilization of bed resources.

The BOR of the Class III hospitals is usually as high as 98%. However, the utilization rate of beds in different departments of a Class III hospital can be very different. West China Hospital (WCH) is typical of such hospitals (see Section 3.1 for more details). The availability of beds for patient hospitalization services is excessive in some units and scarce in others. This discrepancy between bed availability in different departments leads to the overcrowding of some departments and the idleness of other departments. This internal imbalance has further worsened the shortage of hospital resources.

A possible solution to this problem is to allocate the number of beds among different departments in such a way as to increase the utilization rate of beds and alleviate the shortage of bed resources as much as possible. There has been considerable research into the allocation of bed resources. The study of bed resource allocation can be divided into two main approaches.

One approach is to assign the optimal number of beds in a single department or care units, such as surgery units [5], intensive care units [6, 7], and obstetrics departments [8]. For instance, Akkerman and Knip [5] planned the optimal number of beds for cardiac surgery with the goal of reducing patient waiting time. Oerlemans et al. [9] sent an online questionnaire to all ICU physician members in 90 hospitals of the Dutch Society for Intensive Care, the results of which can be used to improve decision-making regarding allocation of ICU resources. Devapriya et al. [10] proposed the strategic bed analysis model, which is a discrete-event simulation model created after a thorough analysis of patient flow and data from Geisinger Health Systems (GHS). Ridge et al. [11] investigated the problem of hospital bed planning in the intensive care unit. Romanin-Jacur and Facchin [12] studied the ward planning of the intensive surgical department and the pediatric semiintensive care unit.

Another approach to the problem focuses on the number of hospital beds throughout the whole hospital. For example, Akcali et al. [13] planned the best use of hospital beds in the entire hospital with the goal of minimizing the total cost. Utley et al. [14] determined the reasonable number of beds for elective patients in the whole hospital in the case of a very low rate of patient cancellation. However, there is little research on the allocation of beds among different departments or wards.

A range of operational research (OR) methods have been developed and applied to problems of healthcare resource allocation [15], especially bed resources. These methods include queuing theory [16, 17], simulation [18, 19], goal programming [20, 21], and mathematical programming [22–24]. Most of the current research uses a single method for each study. However, the premise of queuing theory is based on very strong assumptions, and it is difficult to apply. Mathematical programming can flexibly add constraints and change the objective function according to specific conditions, and it is more popular. Therefore, a hybrid of different methods is more conducive to solving the problem. Simulation and mathematical programming have become increasingly popular approaches to allocate resources in health care. Tontarski [25] utilized simulation-based optimization and mathematical programming for solving complex nurse-scheduling problems. Studies on the bed allocation problem using mathematical programming, especially combined with simulation, are relatively few.

In short, research into bed allocation mostly focuses on the study of the number of beds in a single department. The problem of how to allocate beds in different departments has not been fully studied. Furthermore, most of the current research on bed allocation is based on a single method such as queuing theory [26] or simulation [27]. There are few hybrid models which integrate data analysis, simulation, and mathematical programming. We propose a solution to the bed allocation problem at an operational level. We develop a data-driven hybrid three-stage framework incorporating data analysis, simulation, and mixed integer programming (MIP) to determine the optimal bed allocation strategy. The first stage is to select departments for allocation according to the relationship between the number of beds and the BOR and have this result approved by hospital management. In the second stage, we used a simulation model to calculate the BOR for different numbers of hospital beds. We thus derive the functional relationship between the two variables. The third stage is to find the best number of beds in five departments using a MIP model. Our study aims to alleviate shortage of beds by balancing the utilization of existing beds without increasing the number of beds in a large hospital in China. Overall, the contributions of this study are as follows:

1. Our framework is data driven, making the allocation strategy more rational. (i) Using real data from the hospital, data analysis is used to determine the optimal department needs to allocate beds; (ii) the simulation model is used to simulate the corresponding BOR under different bed allocation scenarios. The relationship between the hospital beds and the BOR is derived from the data, thereby providing data-driven personalized constraints for each department.
2. The objective function of the MIP model is tailored to Chinese needs. Because the beds in large hospitals in China have been overloaded, we are not blindly reducing the BOR of beds but are keeping the number of beds in a reasonable range.
3. This paper provides a general framework for the allocation of beds in the Chinese context. Different hospitals can modify the objective function of the model and appropriately add or reduce constraints according to their needs. Our model provides a reference for hospital management to effectively manage hospital bed resources.

The rest of the paper is organized as follows. In Section 2, we briefly introduced the background of the case hospital (WCH) and the process of the data-driven hybrid three-stage framework. Taking WCH as a case study, we applied the framework proposed in Section 2 to WCH, and these analyses of the framework are shown in Section 3. In Section 4, we discuss the results of the paper. Finally, Section 5 concludes the paper and indicates some directions for future research.

## 2. Materials and Methods

### 2.1. Study Hospital

**Figure 1:**
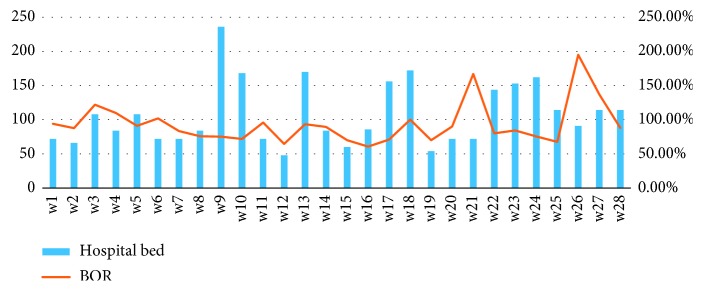
Hospital beds and BOR of 28 departments in West China Hospital.

From a macro perspective, the overall BOR in China's tertiary hospitals is very high. However, at an individual level, the availability of hospital beds in different departments in individual hospitals is uneven. This phenomenon exists in almost all of the tertiary hospitals in China. It is particularly serious in WCH, a tertiary hospital which is located in Chengdu, Sichuan province. In order to rationally manage beds, the Admission Service Center (ASC), a bed planning organization, was established in 2011. It manages 2956 beds and 28 specialized care departments. After our survey and data analysis (Figure 1), we found that the allocation of beds among the 28 departments is not balanced. For example, the BOR of W3 is as high as 122%, while others, such as W12, are only 64.70%. The reason why the BOR is over 100% is that the extra beds are involved in the calculation process. When the number of inpatients exceeds the number of fixed beds, the hospital will add additional beds to meet the demand, and these beds are often arranged in the corridor. This imbalance further leads to inefficiency and waste of hospital bed space, which in turn exacerbates the shortage of hospital beds.

We focus on balancing the BOR of departments by redistributing beds to improve the utilization rate of resources. This study takes WCH as an example to provide a feasible solution for the shortage of hospital beds in large hospitals.

### 2.2. Data Collection

This study used data from the Hospital Information System of the ASC for the period from January 1 to December 31, 2013. It includes the time of each patient admission and discharge, demographic information, and department information and has a total of 243,685 admission registrations and 167,843 discharge records.

### 2.3. Methods

**Figure 2:**
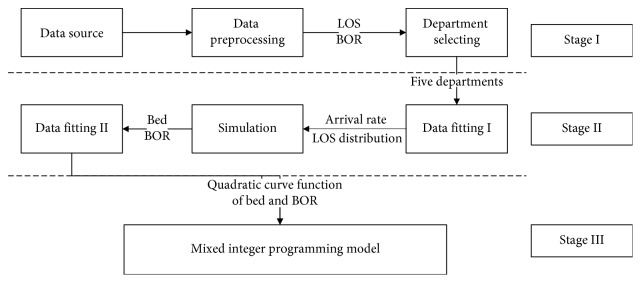
Methodology: process of the data-driven hybrid three-stage framework.

The aim of this research is to balance the BOR of each department by allocating the hospital beds to departments, using a fixed number of existing beds, and keeping the bed utilization rate of each department at a reasonable level. Hence, we proposed a data-driven hybrid three-stage framework to solve this problem. The overall approach is shown in Figure 2.

1. Stage I (data preliminaries): we selected the key departments by analyzing their current BOR and number of beds.
2. Stage II (construction of constraint conditions): Simio software [28] was used to establish a simulation model to obtain the different scenarios of the beds and corresponding BOR. We then determined the relationship between the BOR and the number of beds through data fitting, which is one of the constraints of the Stage III.
3. Stage III (construction of model): we established a MIP model to minimize the gap in BOR among different departments. We applied the genetic algorithm (GA) to solve this model, since GA is one of the best tools for satisfactory solution with advantages like good convergence, low computational complexity, high robustness, and so forth [29].

#### 2.3.1. Stage I: Data Preliminaries

*(1) Calculation of Length of Hospital Stay*. Length of hospital stay (LOS) indicates the number of days the patient spent in a hospital bed. We made the assumption that the LOS can be considered as a constant [30]. We have got 243,685 admission records and 167,843 discharge records form ASC. The LOS is calculated as the discharge date for each patient minus the date of admission registration in the ASC. The sum of the days of all hospitalized patients is an important parameter for calculating the BOR. This paper uses the data from 2013 1/1 to 2013/12/31. We divided the patients into three types by the discharge date as follows and calculated the LOS in 2013 for each group.

- Type I: patients discharged during 2013. Those patients registered in the ASC during or before 2013. Their actual LOS during the 2013 is called LOS1. For example, the actual LOS of the patient who registered before 2013 is discharge date minus January 1, 2013.
- Type II: those patients who left the hospital in 2014, who registered before 2013 or during 2013. Their LOS equals to December 1, 2013, minus registered date or December 31, 2013, minus January 1, 2013, which is named LOS2.
- Type III: because some discharge data are missing, there are patients who are recorded as having been discharged from hospital in 2013 but had no admission records. These patients cannot be ignored. Hence, the number of Type III patients is the total number of discharged patients in one department in 2013 minus the number of Type I. We cannot directly calculate the LOS of Type III patients, so in this study, we used the median of the LOS of Type I patients as the value for the LOS of Type III patients, which we called LOS3.

*(2) The Calculation of BOR*. We calculated each department's BOR according to the following formulas:

(1) $$\begin{array}{c} \text{all patients' LOS}=\text{LOS}1+\text{LOS}2+\text{LOS}3, \end{array}$$

(2) $$\begin{array}{c} \text{BOR}=\frac{\text{all patients' LOS}}{\text{LOS that all beds can provide}}. \end{array}$$

#### 2.3.2. Stage II: Construction of Constraint Conditions

The Simio software was used to build simulation models using data from different departments. Changes in the number of beds, the number of hospitalized patients, and the LOS of patients with different bed numbers were simulated and used to calculate different BOR indexes. The relationship between hospital bed numbers and BOR was an important constraint condition in building the mixed integer programming model in Stage III. It proceeds in three steps:

1. We used the EASY-FIT [31], a professional data fitting software, to fit the patient's arrival distribution and LOS distribution of each department
2. The fitted arrival distribution and LOS distribution were used as the input to the simulation model, which identified the relationship between the number of beds and the BOR
3. We fitted the relationship between the number of beds and the BOR via IBM SPSS Statistics V21 and obtained their functional relationship

#### 2.3.3. Stage III: Construction of the Model

In order to thoroughly understand the hospital bed allocation problem, it is necessary to describe the characteristics of the problem in order to implement them in an appropriate mathematical model. We define parameters and variables of the model:

1. Parameters
2.  *K*_*ij*_: the ward type *j* for department *i*. There are three inpatient ward types.
   
*K*_*i*1_: the number of single-bed wards in department *i*.
   
*K*_*i*2_: the number of double-bed wards in department *i*.
   
*K*_*i*3_: the number of three-bed wards in department *i*
3.  *L*_*i*_: lower bound of number of beds in department *i*.
4.  *U*_*i*_: upper bound of number of beds in department *i*.
5.  *C*_*t*_: the total number of beds of all the departments.
6.  BOR_*i*_(*C*_*i*_): the BOR of department *i*, when the number of beds of department *i* is *C*_*i*_. The relationship functions between BOR_*i*_(*C*_*i*_) and *C*_*i*_ of department *i* can be obtained from the results of Stage II. Here, we assume that the two variables are quadratic functions.
7. Variable
8.  *C*_*i*_: the number of beds of department *i* and *C*_*i*_=*K*_*i*1_+2*K*_*i*2_+3*K*_*i*3_; *C*_*i*_ is a positive integer.
9.  There are *n* departments in WCH, but *i* departments (*i* ∈ 1,2,…, *n*) need to be allocated beds. Our main decision variable is *C*_*i*_, which represents the number of beds for department *i*. Our goal was to balance the BOR of each department; hence, our objective function is to minimize the total gap between BOR of each department and their average BOR. We developed a MIP model as follows:
10. Objective function

(3) $$\begin{array}{c} \min\left(Z\right)=\overset{n}{\underset{i=1}{\sum }}\left|{\text{BOR}}_{i}\left(C_{i}\right)-\left(\overset{n}{\underset{i=1}{\sum }}\frac{{\text{BOR}}_{i}\left(C_{i}\right)}{\text{n}}\right)\right|. \end{array}$$

(4) Constraints

(4) $$\begin{array}{c} {\text{BOR}}_{i}\left(C_{i}\right)=\beta_{i}+a_{i}C_{i}+b_{i}C_{i}{}^{2},\;i=1,2,\ldots n, \end{array}$$

(5) $$\begin{array}{c} \overset{n}{\underset{i=1}{\sum }}C_{i}=C_{t},\;i=1,2,\ldots n, \end{array}$$

(6) $$\begin{array}{c} L_{i}\leq \text{Ci}\leq U_{i},\;i=1,2,\ldots ,n, \end{array}$$

(7) $$\begin{array}{c} C_{i}=K_{i1}+2K_{i2}+3K_{i3},\;i=1,2,\ldots ,n, \end{array}$$

(8) $$\begin{array}{c} K_{ij}\geq 2,\;i=1,2,\ldots ,n,\;j=1,2,3. \end{array}$$

Because of the imbalance between different specialty care departments, we aimed to balance the BOR of various departments without adding extra beds. In objective function (3), (∑_*i*=1_^*n*^BOR_*i*_(*C*_*i*_))/*n* is the average BOR of *n* departments and *Z* is the sum of the gap between the BOR of the *n* departments and the average of their BOR. The purpose of function (3) is to minimize the total gap in BOR between different departments and average BOR.

The constraint described by function (4) means the functional relationship between BOR and the number of beds, which is calculated in Stage II. Here, we assume that the two are quadratic functions: BOR_*i*_(*C*_*i*_)=*β*_*i*_+*a*_*i*_*C*_*i*_+*b*_*i*_*C*_*i*_^2^, where *β*_*i*_ is a constant and *a*_*i*_ and *b*_*i*_ are coefficients (for more details, see Section 3.2); function (5) means that the total number of beds of the *n* departments is constant. Function (6) ensures that there will be an upper limit and a lower limit for the beds in each department. Functions (7) and (8) impose restrictions on the ward type and patient gender. Each ward has either one, two, or three beds. The distinction between male and female wards, and the number of ward types in each department should not be less than two. For example, a single-bed ward has at least two wards so that a male patient can live in one room and a female patient can live in another single room. The other two ward types have the same conditions. Both male and female patients can decide which type of ward to live in, ensuring the fair treatment of patients.

## 3. Results

### 3.1. Stage I: Data Preliminaries

The LOS of 28 departments can be calculated by function (1), and the BOR of the 28 departments can be calculated by function (2).  Table 2 shows the number of beds and BOR in 28 departments of the current WCH. The BOR varies from 60.7% to 195% among the 28 departments. Some literature indicates [32, 33] that the optimal range for BOR is between 85% and 90%. Based on this estimation, we divided these departments into three groups:

1. Group A: BOR is less than 85%. For example, W9 owns 236 beds, but its BOR is only 75.2%.
2. Group B: BOR is greater than 90%. For example, the BOR of W6 reaches as high as 102%, but it only has 72 beds.
3. Group C: BOR is between 85% and 90%. Their bed numbers and BOR are within the normal range, compared to groups A and B.

It is clear that there are serious imbalances in BOR between departments. To solve this problem, we interviewed a hospital manager, three other managerial assistants, and medical physicians of the ASC. We choose five departments (W9, W10, W19, W6, and W27) from groups A and B to solve the problem of bed allocation by applying the framework mentioned in Section 2.3.

### 3.2. Stage II: Construction of Constraint Conditions

After perprocessing the data and selecting the departments, we fitted the distribution of the patient arrival rate and LOS of the five departments using EASY-FIT, and the results are shown in Table 3.

**Figure 3:**
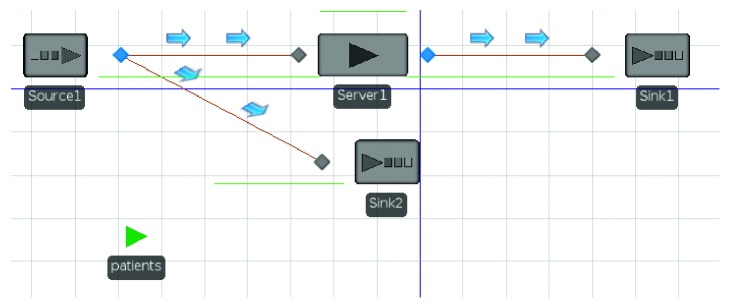
Simulation model of the W9.

We obtained the distribution of arrival rates for all five departments. The fitting of LOS is not ideal. Five departments do not display any distribution. We took the median of the LOS as their distribution. We used W9 as the example from which we can build the simulation model.  Figure 3 describes the simulation model of W9 in Simio.

We set up a patient entity, called Patients, and a patient source called Source1, in the Simio software. We let Source1 associate with Patients and set the arrival rate to obey the Johnson distribution (0.025, 0.803, −8.16, 85.98).

We built a Server1 to represent beds. Its Service capacity was set to the current number of beds (236), and service time was set to 10 days. We calculated the proportion of hospitalizations (43%) based on the number of hospital admissions (12712) and discharges (5478).

There are two leaving routes in the simulation model, namely, Sink1 and Sink2. Sink1 represents the event that a patient leaves the hospital after being served by Server1. The weight from Source1 to Server1 is 43%. Sink2 indicates that a patient who was not admitted to the hospital left the hospital directly through Sink2; the weight from Source1 to Sink2 is 57%.

In order to validate the model, we run the model for a simulated year. The result was that the total number of discharges was 5984, and the BOR was 69.5%. Compared with real data, the difference is 9.2% and 7.5%, respectively. The error was acceptable. Then, we set different parameters for the Server1 and calculated the BOR for different scenarios.

Because W9 belongs to group A, we needed to reduce the number of beds and increase the BOR. We should therefore reduce the number of beds in Server1. In Table 4, we list the number of beds and the corresponding BOR situation for W9. The results of the other four departments are presented in Tables 5–8.

**Figure 4:**
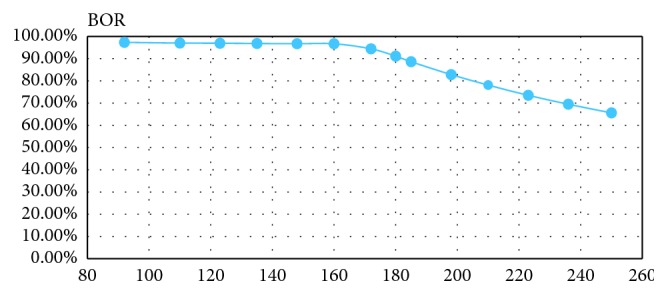
BOR curve for different numbers of beds. The abscissa is the number of beds, and the ordinate is BOR.

We used the number of beds as an independent variable and the BOR as the dependent variable based on the results in Table 4. The graph of BOR changing with the number of beds is shown in Figure 4. It is difficult to intuitively obtain the relationship equation between the two from the graph, so we selected eight kinds of curve functions—linear, logarithm, quadratic, composite, power, growth, exponential, and logistic—with which we can attempt to fit their functional relationships. We used the value of *R*^2^ to determine which relationship function between bed and BOR of W9 had the best fit (Table 9). Since the *R*^2^ value of the quadratic function was the best, at 0.977, we decided that the quadratic function best describes the relationship between the bed numbers and the BOR in W9. We can derive the functional relationship between BOR_1_(*C*_1_) and the bed *C*_1_ from Table 9; the equation is: BOR_1_(*C*_1_)=0.721+0.004*C*_1_ − 0.0000195*C*_1_^2^.

Similar to the analysis process of W9, we obtained the quadratic functional relationships between the BOR and the bed number of the other four departments (Tables 10–13). Hence, the relationship function between beds and BOR of department *i* is expressed as follows:

(9) $$\begin{array}{c} {\text{BOR}}_{i}\left(C_{i}\right)=\beta_{i}+a_{i}C_{i}+b_{i}C_{i}{}^{2}, \end{array}$$

where BOR_*i*_(*C*_*i*_) represents the bed occupancy rate of department *i*, *C*_*i*_ is the number of beds in department *i*, *β*_*i*_ is a constant, and *a*_*i*_ and *b*_*i*_ are coefficients. We have obtained five equations, respectively, for five departments. They are used as constraints for the mixed integer programming model in Stage III.

### 3.3. Stage III: A Mixed Integer Programming Model

After data analysis and simulation in the first two stages, we obtained the parameter values in equations (3)–(8), including the five selected departments and established the quadratic function relationship between the bed numbers and the BOR. We then applied these parameters to equations (3)–(8) to solve the model. The specific MIP model is as follows:

  Objective function

(10) $$\begin{array}{c} \min\left(Z\right)=\overset{5}{\underset{i=1}{\sum }}\left|{\text{BOR}}_{i}\left(C_{i}\right)-\left(\overset{5}{\underset{i=1}{\sum }}\frac{{\text{BOR}}_{i}\left(C_{i}\right)}{5}\right)\right|. \end{array}$$

  Constraints

(11) $$\begin{array}{c} {\text{BOR}}_{1}\left(C_{1}\right)=0.721+0.004C_{1}-0.0000195C_{1}^{2}, \end{array}$$

(12) $$\begin{array}{c} {\text{BOR}}_{2}\left(C_{2}\right)=0.895+0.003C_{2}-0.00002854C_{2}^{2}, \end{array}$$

(13) $$\begin{array}{c} {\text{BOR}}_{3}\left(C_{3}\right)=0.687+0.008C_{3}-0.000049323C_{3}^{2}, \end{array}$$

(14) $$\begin{array}{c} {\text{BOR}}_{4}\left(C_{4}\right)=0.901+0.003C_{4}-0.000020066C_{4}^{2}, \end{array}$$

(15) $$\begin{array}{c} {\text{BOR}}_{5}\left(C_{5}\right)=0.911+0.012C_{5}-0.0003054C_{5}^{2}, \end{array}$$

(16) $$\begin{array}{c} \overset{5}{\underset{i=1}{\sum }}C_{i}=644, \end{array}$$

(17) $$\begin{array}{c} 92\leq C_{1}\leq 210, \end{array}$$

(18) $$\begin{array}{c} 68\leq C_{2}\leq 138, \end{array}$$

(19) $$\begin{array}{c} 57\leq C_{3}\leq 140, \end{array}$$

(20) $$\begin{array}{c} 114\leq C_{4}\leq 182, \end{array}$$

(21) $$\begin{array}{c} 30\leq C_{5}\leq 182, \end{array}$$

(22) $$\begin{array}{c} C_{i}=K_{i1}+2K_{i2}+3K_{i3},\;i=1,2,3,4,5, \end{array}$$

(23) $$\begin{array}{c} K_{ij}\geq 2,\;i=1,2,3,4,5,\;j=1,2,3. \end{array}$$

Constraints (11)–(15) are quadratic functions of the number of beds and the BOR of the five departments. After we obtain the number of beds in each department, we can calculate their BOR by formulas (11)–(15). Constraint (16) states the total number of beds in five departments. Constraints (17)–(21) limit the upper bound and lower bound on bed numbers of each department. Constraints (22) and (23) restrict the ward type and patient gender. There are three ward types for each department in WCH. So, the number of beds in department *i* is the sum of the total number of beds from those three types. In order to distinguish the male and female wards, the number of each department type must be a positive integer and should not be less than two. We used the genetic algorithm [34] to solve the MIP model. The genetic algorithm is run on a personal computer with an Intel® Core™ i7-7700 CPU, a 3.60 GHz z Intel processor, and 8.0 GB RAM. The elapsed time is 77.652611 seconds.

**Figure 5:**
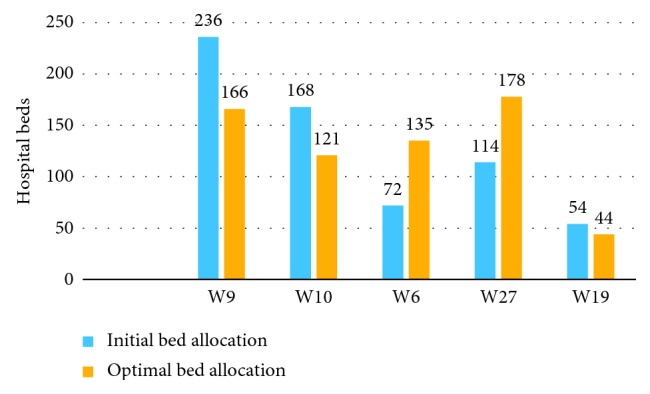
Initial bed allocation strategy and optimal bed allocation strategy.

**Figure 6:**
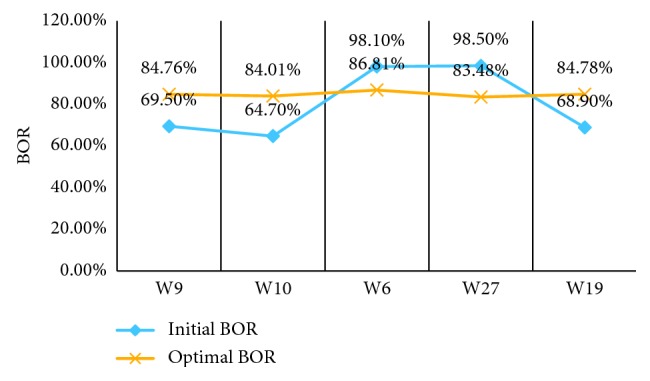
Results of initial BOR and optimal BOR.

We analyzed the results from three aspects:

1. Initial bed allocations and optimal bed allocations based on our model: as shown in Figure 5, the blue histograms represent the Initial bed allocation, which is the current hospital bed number. The optimal bed allocations from our model are represented by the yellow histograms. Figure 5 shows the optimal bed allocation strategy: the bed of the W9 reduces from 236 to 166, W10 from 168 to 121, and W19 from 54 to 44; W6 increases from 72 to 135 and W27 from 114 to 178.
2. Initial BOR and optimal BOR: Figure 6 shows the corresponding BOR after optimization. The W9 increases from 69.5% to 84.76%, W10 from 64.7% to 84.01%, W19 from 68.9% to 84.78%; on the contrary, W6 decreases from 98.1% to 86.81% and W27 from 98.5% to 83.48%. Blue lines represent the original BOR, and yellow lines represent the optimal BOR. We found that the maximum and minimum BORs are 98.1% and 64.7%. The maximum difference of BOR is 33.8% but change to 2.80% after optimization.
3. Objective function value: our objective value, *Z*, represents the imbalance degree of bed utilization between various departments. For baseline bed allocation, the initial value of the objective function *Z* is as high as 0.7344. After optimization, the optimal value of *Z* is 0.0409, indicating that our optimization reduced the severity of the imbalance.

Finally, we can get a combination of beds in different wards based on the number of optimal beds (Table 14). In formula (22), *C*_*i*_ depends on the value of *K*_*ij*_, that is to say, the combinations of *K*_*ij*_ produce different *C*_*i*_ values. For example, the optimal number of beds for W9 determined by our model is 166 (*C*_1_=166); there are many combinations for single-bed wards (*K*_11_), double-bed wards (*K*_12_), and triple-bed wards (*K*_13_), such as (26, 40, 20), (22, 42, 20), and (36, 20, 30). This means that W9 can provide 26 single-bed wards, 40 double-bed wards, and 20 triple-bed wards; 22 single-bed wards, 42 double-bed wards, and 20 triple-bed wards; or 36 single-bed wards, 20 double-bed wards, and 30 triple-bed wards; and so on.

## 4. Discussion

Many tertiary hospitals in China are facing the same problem as WCH, with respect to the imbalance in the utilization of bed resources in different departments. The availability of beds for hospital care is excessive in some cases and scarce in others. This phenomenon has caused many problems for hospitals. For example, some hospital wards are always overcrowded, while others are underloaded. Some scheduled patient admissions are delayed or even transferred to other hospitals, and some patients are hospitalized in inappropriate wards which are unsuited to their pathologies, with the risk of a lower quality care and a greater chance of infection [35].

To relieve this imbalance, we propose a data-driven hybrid three-stage framework combining multiple methods to produce a feasible bed allocation strategy since it is difficult to allocate beds among all 28 departments in the whole WCH. We selected five departments (W9, W10, W6, W27, and W19) through data analysis and survey interview. W9, W10, and W19 are departments that have many beds with a low BOR while W6 and W27 have few beds with high BOR. For Stage II, we developed a generic discrete-event simulation model. We fitted the relationship function between BOR and beds of each wards via the simulation model. In Stage III, we developed a MIP model to minimize the imbalance in BOR. The results of Stage II are incorporated into the MIP model as one of the key constraints. We also considered other constraints, such as ward types (single, double, and three-bed wards) and upper and lower bounds on the number of beds.

Our data-driven hybrid three-stage framework produces a flexible allocation strategy for hospital bed management. Our research helps to improve the utilization of medical resources and the quality of medical services by balancing bed numbers and BOR between different departments. Our model may be applied in two ways. Firstly, it can be extended to other wards with different arrival rates and LOS distribution. Secondly, our study can provide a reference for dealing with the problem of hospital bed capacity to other large general hospitals in China. Our research provides a common framework for hospital bed allocation, so other departments or hospitals can follow our three-stages framework to realize their allocation of beds. Because the data of each hospital and the actual situation are different from those of WCH, different constraints or objective functions may be generated. For example, other hospitals can use data analysis to screen the departments that need to allocate beds; then, they can follow the method described in Stage II to fit the functional relationship between BOR and bed. Different hospitals may have different functional relationships because of their different data. Finally, hospitals can redesign the model with more personalized objective functions and constraints according to their own actual situation.

## 5. Conclusions

We focused upon the problem of allocating beds among different departments in a hospital. We took a large public hospital in China, WCH, as a case study. To relieve imbalances in BOR between departments, we proposed a three-stage framework. In the first stage, we collected data and identified departments of interest. In the second stage, we identified the functional relationship between the number of beds and the BOR. The third-stage MIP model provides the best number of bed allocations for different departments. It has proven to be a feasible method to ease the shortage of beds.

Our research is based on real data, and hospital managers can draw upon the results of this study to solve the bed occupancy and capacity problem. The three-stage framework can help bed managers adjust the allocation of beds in a timely and dynamic manner. This approach can be applied to the majority of other hospitals and may serve as a starting point for the development of allocation models for other service industries with similar conditions, such as the allocation of beds or room types in hotels.

Future study can consider the following two aspects: since this is the initial stage of bed allocation, the strategy can be extended to more departments. In addition, the practice will be a good reference for other large general hospitals in China. More factors may be considered for inclusion in the MIP model such as other ward resources (nurses and doctors), infectious patients, and the undesirability of mixed-sex rooms.

## Figures and Tables

**Table 1:** Hospital beds, hospitalization, and BOR of China's large tertiary hospitals in 2015 and 2016.

Hospital level	Hospital beds		Hospitalization		BOR	
	2015	2016	2015	2016	2015 (%)	2016 (%)
Class III	2,047,819	2,213,718	68,290,000	76,860,000	98.80	98.80
Class II	2,196,748	2,302,887	71,210,000	75,700,000	84.10	84.20
Class I	481,876	517,837	9,650,000	10,390,000	58.80	58

**Table 2:** Department information summary.

A	Department	W7	W8	W9	W10	W12	W15	W16	W17	W19
	Beds	72	84	236	168	48	60	86	156	54
	BOR (%)	83.4	75.7	75.2	71.8	64.7	70	60.7	71.1	70
	Department	W24	W23	W25						
	Beds	162	153	114						
	BOR (%)	75.60	84.40	67.60						

B	Department	W1	W3	W4	W5	W6	W11	W13	W18	W21
	Beds	72	108	84	108	72	72	170	172	72
	BOR (%)	94.0	122	110	91	102	95.8	93.5	100	167
	Department	W26	W27	W26						
	Beds	91	114	91						
	BOR (%)	195	137	195						

C	Department	W1	W2	W20	W28					
	Beds	84	66	72	114					
	BOR (%)	89.6	87.7	90	88.1					

**Table 3:** Results of arrival rate distribution and LOS.

Departments	Department type	Arrival rate	LOS
W6	B	Johnson SB (0.289, 0.983, −3.34, 35.11)	Median = 9
W9	A	Johnson SB (0.025, 0.803, −8.16, 85.98)	Median = 10
W10	A	Uniform (−0.72, 43.75)	Median = 12
W19	A	Uniform (−3.2, 92.36)	Median = 10
W27	B	Uniform (−3.2, 92.36)	Median = 5

**Table 4:** Beds and corresponding BOR of W9 in the simulation model.

Beds	Discharge	BOR (%)
92	3266	97.3
110	3896	97
123	4351	96.9
135	4771	96.8
148	5226	96.7
160	5646	96.68
172	5926	94.4
180	5982	91.1
185	5984	88.6
198	5984	82.8
210	5984	78.1
223	5984	73.5
236^*∗*^	5984	69.5
250	5984	65.6

^*∗*^The current number of hospital beds of W9.

**Table 5:** Beds and corresponding BOR of W10.

Beds	Discharge	BOR (%)
68	2011	97.2
78	2301	97
88	2585	96.6
98	2865	96.1
108	3145	95.7
113	3201	93.1
118	3216	89.6
128	3246	83.4
138	3276	78
148	3299	73.3
158	3306	68.8
168	3306	64.7
178	3306	61.1

**Table 6:** Beds and corresponding BOR of W6.

Beds	Discharge	BOR (%)
57	2279	98.6
62	2474	98.4
72	2864	98.1
82	3254	97.8
95	3756	97.4
100	3931	96.9
105	4106	96.4
110	4281	96
115	4438	95.1
120	4516	92.8
125	4538	89.5
130	4538	86.1
140	4538	80

**Table 7:** Beds and corresponding BOR of W27.

Beds	Discharge	BOR (%)
114	8196	98.50
130	9332	98.30
145	10345	97.70
149	10482	96.37
152	10527	94.90
160	10640	91.10
168	10674	87
175	10681	83.60
182	10688	80.40
190	10696	77.10
205	10711	71.60
220	10726	66.80

**Table 8:** Beds and corresponding BOR of W19.

Beds	Discharge	BOR (%)
30	995	97.2
35	1153	97
36	1189	96.8
39	1225	93
38	1165	90.3
40	1258	92.2
45	1246	80.9
47	1230	77.4
50	1246	73.05
52	1252	70.6
54	1274	68.9

**Table 9:** Relationship between bed numbers and BOR in W9.

Function	Model					Parameters			
	*R* ^2^	F	df1	df2	Sig.	Constant	b1	b2	b3
Linear	0.836	61.326	1	12	0.000	1.253	−0.002		
Logarithm	0.724	31.538	1	12	0.000	2.550	−0.328		
Quadratic	0.977	230.860	2	11	0.000	0.721	0.004	0.0000195	
Composite	0.820	54.831	1	12	0.000	1.367	0.997		
Power	0.704	28.548	1	12	0.000	6.455	−0.392		
Growth	0.820	54.831	1	12	0.000	0.313	−0.003		
Exponential	0.820	54.831	1	12	0.000	1.367	−0.003		
Logistic	0.820	54.831	1	12	0.000	0.732	1.003		

**Table 10:** Relationship between bed numbers and BOR in W10.

Function	Model					Parameters			
	*R* ^2^	F	df1	df2	Sig.	Constant	b1	b2	b3
Linear	0.919	125.130	1	11	0.000	1.298	−0.004		
Logarithm	0.837	56.598	1	11	0.000	2.813	−0.413		
Quadratic	0.973	181.654	2	10	0.000	0.895	0.003	−2.854*E* − 5	
Composite	0.907	107.562	1	11	0.000	1.468	0.995		
Power	0.817	49.021	1	11	0.000	9.578	−0.513		
Growth	0.907	107.562	1	11	0.000	0.384	−0.005		
Exponential	0.907	107.562	1	11	0.000	1.468	−0.005		
Logistic	0.907	107.562	1	11	0.000	0.681	1.005		

*Note.* The higher the *R*^2^, the better the function model. In the W10 model fitting, the fitting degree of the quadratic curve is the best, and the quadratic curve is directly selected. The bed number of W10 is *C*_2_, and the bed utilization rate is BOR_2_ (*C*_2_). According to the estimated value of the parameter, BOR_2_(*C*_2_)=0.895+0.003*C*_2_ − 0.00002854*C*_2_^2^.

**Table 11:** Relationship between beds and BOR in W6.

Function	Model					Parameters			
	*R* ^2^	F	df1	df2	Sig.	Constant	b1	b2	b3
Linear	0.664	21.741	1	11	0.001	1.118	−0.002		
Logarithm	0.558	13.883	1	11	0.003	1.616	−0.147		
Quadratic	0.956	107.465	2	10	0.000	0.687	0.008	−4.932*E* − 5	
Composite	0.645	19.978	1	11	0.001	1.140	0.998		
Power	0.539	12.849	1	11	0.004	1.966	−0.161		
Growth	0.645	19.978	1	11	0.001	0.131	−0.002		
Exponential	0.645	19.978	1	11	0.001	1.140	−0.002		
Logistic	0.645	19.978	1	11	0.001	0.877	1.002		

*Note*. The higher the *R*^2^, the better the function model. When the W6 model is fitted, it has the same outcome as W9. The quadratic curve is also selected. The bed number of W6 is *C*_3_, and the bed rate is BOR_3_ (*C*_3_). According to the estimated value of the parameter, BOR_3_(*C*_3_)=0.687+0.008*C*_3_ − 0.000049323*C*_3_^2^.

**Table 12:** Relationship between beds and BOR in W27.

Function	Model					Parameters			
	*R* ^2^	F	df1	df2	Sig.	Constant	b1	b2	b3
Linear	0.932	137.447	1	10	0.000	1.446	−0.003		
Logarithm	0.882	74.410	1	10	0.000	3.673	−0.550		
Quadratic	0.967	131.496	2	9	0.000	0.901	0.003	−2.007*E* − 5	
Composite	0.964	119.131	2	9	0.000	1.094	0.000	−2.585*E* − 6	−3.039*E* − 8
Power	0.924	121.995	1	10	0.000	1.720	0.996		
Growth	0.866	64.725	1	10	0.000	24.281	−0.655		
Exponential	0.924	121.995	1	10	0.000	0.542	−0.004		
Logistic	0.924	121.995	1	10	0.000	1.720	−0.004		

*Note*. The higher the *R*^2^, the better the function model. In the W27 model fitting, the fitting of the quadratic curve is the best, and the quadratic curve is selected. The bed number of W27 is *C*_4_, and the bed utilization rate is BOR_4_ (*C*_4_). According to the estimated value of the parameter, BOR_4_(*C*_4_)=0.901+0.003*C*_4_ − 0.000020066*C*_4_^2^.

**Table 13:** Relationship between beds and BOR in W19.

Function	Model					Parameters			
	*R* ^2^	F	df1	df2	Sig.	Constant	b1	b2	b3
Linear	0.947	159.652	1	9	0.000	1.450	−0.014		
Logarithm	0.915	96.619	1	9	0.000	3.011	−0.579		
Quadratic	0.966	114.721	2	8	0.000	0.911	0.012	−3.054*E* − 5	
Composite	0.964	108.277	2	8	0.000	1.095	0.000	−5.576*E* − 5	−1.687*E* − 6
Power	0.944	151.827	1	9	0.000	1.736	0.983		
Growth	0.908	88.465	1	9	0.000	11.291	−0.695		
Exponential	0.944	151.827	1	9	0.000	0.552	−0.017		
Logistic	0.944	151.827	1	9	0.000	1.736	−0.017		

*Note*. The higher the *R*^2^, the better the function model. In the W19 model fitting, the fitting of the quadratic curve is the best, and the quadratic curve is selected. The bed number of W19 is *C*_5_, and the bed utilization rate is BOR_5_ (*C*_5_). According to the estimated value of the parameter, BOR_5_(*C*_5_)=0.911+0.012*C*_5_ − 0.0003054*C*_5_^2^.

**Table 14:** Optimal number of beds and different combinations of departments.

Department	Optimal number of beds	Different feasible combinations of *K*_*ij*_ : (*K*_*i*1_, *K*_*i*2_, *K*_*i*3_)
W9	166	(*K*_11_, *K*_12_, *K*_13_) : (26, 40, 20), (22, 42, 20), (36, 20, 30),…
W10	121	(*K*_21_, *K*_22_, *K*_23_) : (21, 20, 20), (21, 35, 10), (31, 30, 10),…
W6	135	(*K*_31_, *K*_32_, *K*_33_) : (35, 20, 20), (20, 40, 7), (25, 10, 30),…
W27	178	(*K*_41_, *K*_42_, *K*_43_) : (78, 20, 20), (10, 39, 30), (40, 39, 20),…
W19	44	(*K*_51_, *K*_52_, *K*_53_) : (14, 6, 6), (4, 5, 10), (19, 5, 5),…

## Data Availability

The data used to support the findings of this study are restricted by the West China Hospital in order to protect patient privacy. Data are available from West China Hospital for researchers who meet the criteria for access to confidential data.

## Appendix

General hospitals have been divided into three levels in China, according to the hospital's functions, tasks, facilities, technology, medical services, and scientific management.

Class I hospitals (the number of beds is 100 or fewer): primary hospitals and health centers that provide prevention, medical care, health care, and rehabilitation services directly to communities in a specific population.

Class II hospitals (101 to 500 beds): regional hospitals that provide comprehensive medical and health services to multiple communities and undertake certain teaching and research tasks.

Class III hospitals (also called tertiary hospitals; authors' hospital; more than 501 beds): regional or higher hospitals that provide high-level specialized medical and health services and perform higher education and scientific research tasks in several areas. Tertiary hospitals are further subdivided into three grades based upon the technical strength of the hospital, management levels, equipment conditions, scientific research capabilities, and more. The West China Hospital is one of the highest-grade hospitals among the tertiary hospitals.
